# Retraction Note: Gold Nanoparticles-enabled Efficient Dual Delivery of Anticancer Therapeutics to HeLa cells

**DOI:** 10.1038/s41598-022-15966-1

**Published:** 2022-07-05

**Authors:** Muhammad U. Farooq, Valentyn Novosad, Elena A. Rozhkova, Hussain Wali, Asghar Ali, Ahmed A. Fateh, Purnima B. Neogi, Arup Neogi, Zhiming Wang

**Affiliations:** 1 Institute of Fundamental and Frontier Science, University of Electronic Science and Technology of China, Chengdu, 610054 China; 2 Materials Science Division, Argonne National Laboratory, Argonne, IL 60439 USA; 3 National University of Science and Technology (MISiS), Moscow, 119049 Russian Federation; 4 Nanoscience and Technology Division, Center for Nanoscale Materials, Argonne National Laboratory, Argonne, IL 60439 USA; 5 Department of Physics, International Islamic University Islamabad, Islamabad, 44000 Pakistan; 6 School of Life Science and Technology, University of Electronic Science and Technology of China, Chengdu, 610054 China; 7 Department of Biological Science, University of North Texas, Denton, TX 76203 USA; 8 Department of Physics, University of North Texas, Denton, TX 76203 USA

Retraction of: *Scientific Reports* 10.1038/s41598-018-21331-y, published online 13 February 2018

The Editors have retracted this Article.

Concerns were brought to the attention of the Editors with respect to apparent inappropriate manipulation of the data in seven of the nine panels shown in Figure 7. The Editors no longer have confidence in the data reported in this Article.

Muhammad U. Farooq disagrees with this retraction. Valentyn Novosad and Elena A. Rozhkova agree with this retraction and its wording. Asghar Ali, Ahmed A. Fateh and Zhiming Wang did not respond to correspondence from the Editors about this retraction. Purnima B. Neogi has not explicitly stated whether they agree or disagree with this retraction. The Editors were not able to establish current contact details for Hussain Wali and Arup Neogi.

